# The association between use of electronic media and prevalence of headache in adolescents: results from a population-based cross-sectional study

**DOI:** 10.1186/1471-2377-10-12

**Published:** 2010-02-09

**Authors:** Astrid Milde-Busch, Rüdiger von Kries, Silke Thomas, Sabine Heinrich, Andreas Straube, Katja Radon

**Affiliations:** 1 Institute of Social Paediatrics and Adolescent Medicine, Ludwig-Maximilians-University Munich, Heiglhofstrasse 63, 81377 Munich, Germany; 2 Institute and Outpatient Clinic for Occupational, Social and Environmental Medicine, Unit for Occupational and Environmental Epidemiology & NetTeaching, Hospital of the University Munich, Ziemssenstrasse 1, 80336 Munich, Germany; 3 Department of Neurology, Klinikum Grosshadern, Hospital of the University Munich, Marchioninistrasse 15, 81377 Munich, Germany

## Abstract

**Background:**

Use of electronic media, i.e. mobile phones, computers, television, game consoles or listening to music, is very common, especially amongst adolescents. There is currently a debate about whether frequent use of these media might have adverse effects on health, especially on headaches, which are among the most-reported health complaints in adolescents. The aim of the present study was to assess associations between frequent use of electronic media and the prevalence of different types of headache in adolescents.

**Methods:**

Data were derived from a population-based sample (n = 1,025, ages 13-17 years). Type of headache (i.e. migraine, tension-type headache, unclassifiable headache) was ascertained by standardized questionnaires for subjects reporting headache episodes at least once per month during the last six months. Duration of electronic media use was assessed during personal interviews. Associations were estimated with logistic regression models adjusted for age group, sex, family condition and socio-economic status.

**Results:**

Most of the adolescents used computers (85%), watched television (90%) or listened to music (90%) daily, otherwise only 23% of the participants used their mobile phones and only 25% played with game consoles on a daily basis. A statistically significant association between listening to music and any headache (odds ratio 1.8; 95% confidence interval 1.1-3.1 for 30 minutes per day, 2.1; 1.2-3.7 for 1 to 2 hours per day; 2.0; 1.2-3.5 for 3 hours and longer listening to music per day) was observed. When stratifying for type of headache, no statistically significant association was seen.

**Conclusions:**

Apart from an association between listening to music on a daily basis and overall headache, no consistent associations between the use of electronic media and different types of headache were observed.

## Background

Use of electronic media is very popular amongst German adolescents: almost all families have a television, video players, computers and internet access; listening to the radio or music is widespread amongst almost all adolescents and nearly all adolescents own a mobile phone [1-3]. While girls use mobile phones and music media more frequently than boys, boys use computers, the internet and game consoles more frequently [1-4]. Most studies on this topic report that frequency of usage increases with age [1-3,5] and depends on socio-economic status (SES) [3,6,7].

Excessive use of electronic media is often reported to be associated with long-lasting adverse effects on health like obesity [8,9] or lack of regular exercise [10], increased health-compromising behaviors like smoking or hazardous consumption of alcohol [9,11], increased health complaints [4,12-14] or unspecific symptoms like tiredness, stress, concentration difficulties and sleep disturbances [5,15,16].

One of the most important health problems worldwide is manifest headache [17]: approximately 10% of adolescents suffer from migraine and 15-20% from tension-type headache (TTH) [18-21].

Most studies on associations between use of electronic media and headache have focused on *mobile phone use*, but with a few exceptions [5,22] most of them were done among adults. Controversial results were observed: While some observational studies reported statistically significant associations between the self-reported duration of mobile-phone calls per day or exposure to mobile-phone base stations and headache [5,22-24], experimental studies with controlled exposure duration to radio frequency fields did not find significantly increased headache [25,26]. Regarding the use of *computers*, most studies on adolescents found statistically significant increased prevalences of migraine and TTH [27-30]; only one recent study by Smith et al. did not find such an association [31]. Other studies showed harmful associations between frequent *watching of television *and headache [32], especially migraine [27,28]. An adverse effect of frequently listening to *music *on headache was described by Zarowski et al. who reported that listening to music during nighttime may affect sleep habits and may, therefore, be associated with headache in children and adolescents [32]. Furthermore, the use of *game consoles *(i.e., PlayStation from Sony, Xbox from Microsoft or Wii from Nintendo) may be associated with the prevalence of headache. However, to our knowledge no studies have so far been published that investigated this potential association.

The aims of our investigations were to assess the association between use of different types of electronic media (mobile phones, computer, playing with game consoles, watching television, listening to music) and prevalence of headache and to differentiate associations between different types of headache (migraine, TTH, miscellaneous headache) within one study.

## Methods

The present study was part of the MobilEe project, an epidemiologic investigation of possible effects of exposure to radiofrequency electromagnetic fields on wellbeing in children and adolescents conducted in Bavaria, Germany. It was funded by the German Mobile Telecommunication Research Programme (DMF). MobilEe is described elsewhere in more detail [33]. A population-based sample of adolescents was personally interviewed. In this survey, a broader scale of health conditions was investigated, including a screening question on prevalent headache during the last six months. Adolescents (13 to 17 years), who reported at least one episode of headache per month during the last six months, were invited to answer a questionnaire for a more detailed investigation of type of headache.

The research was approved by the Ethics Committee of the Medical Faculty of the Ludwig-Maximilians-University Munich (285/03). Written informed consent was obtained from the participants' parents and - if participants were older than 14 years - from the adolescents themselves as well.

### Participants

**Figure 1:**
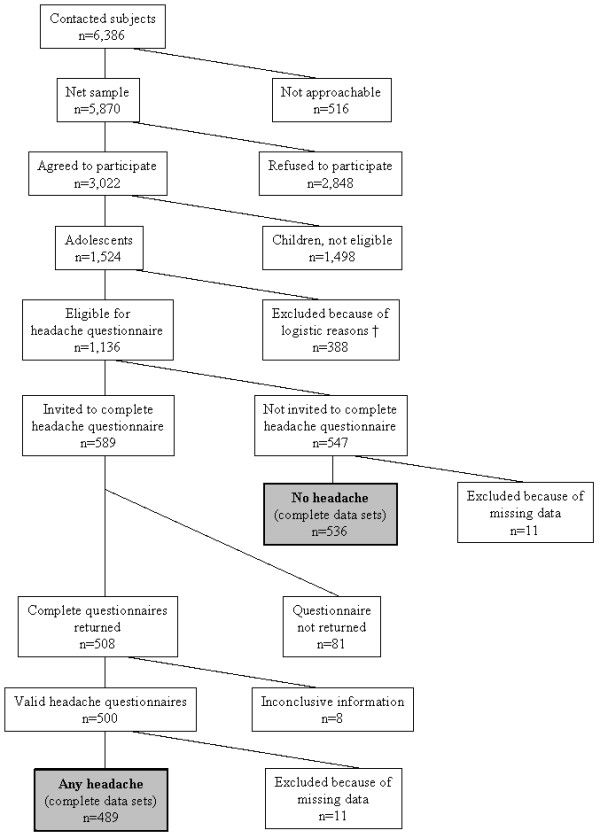
**Flow diagram of subject participation**. Subjects included in the headache study are marked shaded.

Every resident in Germany is forced by law to register at the population registry of his place of residence; taken together these registries list all inhabitants of Germany. Participants in the MobilEe project were randomly drawn from the population registries in four Bavarian cities. Six thousand three hundred and eighty-six children and adolescents were invited to participate in a personal interview: 516 could not be approached and 2,848 refused to participate. From the 3,022 participants with completed interviews, 1,498 were children younger than 13 years and therefore not eligible for the present study. For 388 of the 1,524 remaining adolescents, inclusion in the headache study was not possible, since they had been recruited prior to the conception of the headache substudy, leaving 1,136 adolescents for the present study. Of these 1,136 subjects, 547 reported no headache episodes during the last six months, while 589 subjects reported headache and were therefore invited to complete the headache questionnaires. For 508 of these, the questionnaire was completed, but 8 had to be excluded because of inconclusive information. A further 11 subjects without headache and 11 subjects with valid headache questionnaires had to be excluded because of missing values in the potential socio-demographic confounding variables. This leaves a final study population of 1,025 subjects, of whom 489 had valid headache questionnaires ('any headache' group) and 536 were without any headache ('no headache' group; see Figure 1).

A non-responder analysis with respect to socio-demographic variables revealed that adolescents from families with higher levels of education were more willing to participate in the MobilEe study [33].

### Data collection

Data were collected in public buildings, like city halls or public health departments. Computer-assisted personal interviews took between 15 and 25 minutes to complete. Participants who indicated that they had suffered from headache at least once per month during the last six months were asked to fill in a detailed questionnaire on type of headache.

Validated questions on socio-demographic variables from the German Health Interview and Examination Survey for Children and Adolescents (KiGGS) were used [34]. The definition of the adolescents' SES was based on their own level of education and on the job position of their parents. Level of education was assigned on a scale from 1 point (general secondary school) to 5 points (grammar school). In a first step, parental jobs were classified using the International Standard Classification of Occupations (ISCO-88). Afterwards, the jobs were assigned to the International Socio-Economic Index of Occupational Status [ISEI; [35,36]]. Each ISCO code holds a specific ISEI value, where a high value stands for a job with a high prestige. ISEI values were then divided into quintiles. Level of education and assigned ISEI quintile were added together and revealed the following SES levels: low SES (2-4 points), middle SES (5-7 points), high SES (8-10 points) [see [7]]. For the present analyses, low/middle and high SES were dichotomized.

Earlier studies using MobilEe data [7,37,38] revealed that sex (male, female), age group ('14 years and younger', '15 years and older'), family condition ('complete' if living together with both parents, otherwise 'incomplete') and SES (low/middle, high) are associated with headache and/or with electronic media use and are therefore to be considered as socio-demographic confounding variables in the present study.

Use of mobile phones was assessed with the following item: 'On an average day, how long (in minutes) do you use your mobile phone for voice calls?' with the response categories 'not at all', 'less than 5 minutes', '6 to 15 minutes', '16 to 30 minutes' and 'more than 30 minutes'.

Questions investigating use of the other electronic media were taken from the KiGGS interview [3]: Average daily use of computer/internet, watching television/videos, playing with game consoles and listening to music was assessed using a 5-point response scale ('never', 'approximately 30 minutes', 'approximately 1 to 2 hours', 'approximately 3 to 4 hours', 'more than 4 hours'), separately for weekdays and weekend days. Average use of each electronic media was estimated as weighted means from weekdays and weekend days. An overall media index for use of electronic media was estimated by adding up time spent with computer/internet, television/videos and playing with game consoles [3].

### Headache questionnaire

The headache screening question in the interview ('How often did you experience headache within the last six months?') was part of the symptom checklist developed for the survey of Health Behaviour in School-aged Children [39]. Subjects who indicated that they had suffered from headache at least once per month were given a detailed questionnaire on type of headache, based on the International Classification of Headache Disorders - 2^nd ^edition (ICHD-II) [40].

Any type of migraine and any type of TTH were classified according to the ICHD-II criteria as primary headache disorders. The classifications of pure migraine included the subtypes migraine (with or without aura) and probable migraine according to the strict criteria for adults. Furthermore, probable migraine was classified according to a modified criterion for children with a shorter duration of headache (i.e., attacks between 30 minutes and 72 hours). This criterion was chosen for the sake of comparability with another German study on headache in adolescents [[20]; see also [37,38]] although it does not quite match the conventional ICHD-II criterion for children, which requires attack rates of at least one and up to 72 hours. The classification of pure TTH included the subtypes infrequent episodic TTH, frequent episodic TTH, chronic TTH, probable infrequent episodic TTH, probable frequent episodic TTH and probable chronic TTH. The diagnostic criteria for probable migraine and any probable TTH require agreement with all but one of the respective diagnostic criteria for the migraine or TTH, respectively. Therefore, a double diagnosis of 'migraine *plus *TTH' could arise.

All other subjects with headache who did not match any of these diagnoses for primary headache were considered as miscellaneous headache (MH).

### Statistical analysis

Differences in categorical variables (i.e. media use) were tested by using Cochrane-Armitage tests for underlying trends with ordered categories (i.e. different durations of media use), studying dose-response effects. Multivariate analyses were done using logistic regression models adjusted for age group, sex, family condition and SES as potential socio-demographic confounding variables. Separate regression models for each type of electronic media (mobile phone, computer/internet, television/videos, game consoles, music, overall media index) and each type of headache ('pure migraine', 'pure TTH', 'migraine+ TTH' and 'MH' against no headache) were calculated. Further regression models for each type of headache were calculated, considering all types of electronic media (mobile phone, computer/internet, television/videos, game consoles, music) and socio-demographic variables (age group, sex, family condition, SES). Odds ratios (OR) with 95% confidence interval (CI) for associations between no vs. different durations of media use and six-month prevalence of headache were reported. Calculations were performed with the SAS software package (version 9.1, SAS Institute Inc. Cary, NC, USA).

## Results

### Use of electronic media

Table 1 (first column) shows the distribution of average duration of use of electronic media in the study population. Use of mobile phones appears to be low in the present sample: 77% of the subjects reported little (<5 minutes) or no mobile-phone use per day. However, the vast majority of the adolescents reported watching television/videos and/or listening to music daily and more than 85% of adolescents reported using computer/internet daily. Playing with game consoles on a daily basis was reported with a lower frequency (27%).

**Table 1:** Frequency (%) of electronic-media use, stratified for age group, sex, family condition and socio-economic status.

	Total	Age group		Sex		Family condition		Socio-economic status	
	(n = 1,025)	14 years and younger (n = 472)	15 years and older (n = 553)	Male (n = 500)	Female (n = 525)	Complete (n = 750)	Incomplete (n = 275)	High (n = 581)	Low/middle (n = 444)
**Mobile phone**									
No mobile phone	11.0	16.3	6.5	13.4	8.8	11.3	10.2	11.2	10.9
<5 minutes	65.9	64.2	67.3	68.2	63.6	68.9	57.5	70.2	60.1
6 - 15 minutes	15.6	13.8	17.2	14.2	17.0	14.0	20.0	13.9	17.8
16 - 30 minutes	4.5	3.0	5.9	2.6	6.3	3.5	7.2	3.4	5.9
30 minutes or more	3.0	2.8	3.3	1.6	4.4	2.3	5.1	1.2	5.4
*p†*		***0.0001***		***0.0001***		***0.0001***		***0.0001***	

**Computer/internet**									
Never	13.2	18.9	8.3	12.2	14.1	11.6	17.5	10.7	16.4
30 minutes	33.9	39.6	28.9	31.6	36.0	34.3	32.7	35.8	31.3
1 - 2 hours	38.2	31.6	43.9	39.0	37.5	39.2	35.6	40.6	35.1
3 hours or more	14.7	10.0	18.8	17.2	12.4	14.9	14.2	12.9	17.1
*p*		***0.0001***		***0.0204***		*0.0844*		*0.6160*	

**Television/videos**									
Never	5.4	4.9	5.8	5.6	5.1	4.9	6.6	7.4	2.7
30 minutes	29.8	31.1	28.6	28.8	30.7	30.5	27.6	33.2	25.2
1 - 2 hours	51.4	51.9	51.0	53.6	49.3	53.5	48.4	52.0	50.7
3 hours or more	13.5	12.1	14.6	12.0	14.9	12.0	17.5	7.4	21.4
*p*		*0.4848*		*0.6874*		*0.3370*		***0.0001***	

**Game consoles**									
Never	72.9	64.2	80.1	58.0	86.9	73.6	70.6	76.2	68.2
30 minutes	16.4	22.0	11.6	21.8	11.2	16.3	16.7	14.6	18.7
1 - 2 hours	8.4	11.4	5.8	15.4	1.7	8.7	7.6	7.9	9.0
3 hours or more	2.4	2.3	2.5	4.8	0.2	1.5	5.1	1.2	4.1
*p*		***0.0001***		***0.0001***		*0.0775*		***0.0016***	

**Music**									
Never	8.2	12.1	4.9	10.4	6.1	9.1	5.8	7.7	8.8
30 minutes	29.4	36.0	23.7	32.8	26.1	31.2	24.4	30.6	27.7
1 - 2 hours	30.0	28.8	31.1	27.8	32.2	30.9	27.6	32.4	27.0
3 hours or more	32.4	23.1	40.3	29.0	35.6	28.8	42.2	29.3	36.5
*p*		***0.0001***		***0.0003***		***0.0001***		*0.1830*	

**Overall media index‡**									
Never	1.2	1.5	0.9	1.4	1.0	0.9	1.8	0.9	1.6
30 minutes	2.9	4.0	2.0	1.6	4.2	2.9	2.9	2.9	2.9
1 - 2 hours	28.3	31.6	25.5	23.8	32.6	28.4	28.0	32.9	22.3
3 - 4 hours	32.3	30.3	34.0	32.2	32.4	32.5	31.6	31.5	33.3
5 - 6 hours	23.9	22.0	25.5	27.4	20.6	24.5	22.2	23.2	24.8
7 hours or more	11.4	10.6	12.1	13.6	9.3	10.7	13.5	8.6	15.1
*p*		***0.0072***		***0.0001***		*0.8948*		***0.0009***	

† p values of Cochrane-Armitage tests for underlying trends of media use between categories of socio-demographic variables (younger vs. older, male vs. female, complete vs. incomplete family, high vs. low/middle socio-economic status).

‡ The overall media index was estimated by adding up time spent with computer/internet, television/videos and game consoles.

Age group, sex, family condition and SES were found to be significantly associated with the use of at least one of the electronic media (see Table 1 for a detailed presentation of the associations).

### Prevalence of headache

Among the 489 subjects with prevalent headache out of the 1,025 included subjects (47.7%), pure migraine was found in 42 (4.1%) and pure TTH in 212 (20.7%) of the participants. Any type of migraine plus any TTH was reported by 122 (11.9%) of the subjects. In the remaining 113 (11.0%) subjects with headache, the type of headache could not be classified according to the ICHD-II criteria (MH; see [37] for a detailed analysis of the subtypes of migraine and TTH).

### Associations between use of electronic media and headache

Bivariate trend tests denoted that there may be associations between duration of usage of electronic media and headache (Table 2): Prevalence of any type of headache increased with increasing duration of listening to music (p = 0.0026). After adjusting for age group, sex, family condition and SES as potential confounding variables, this association remained statistically significant (Table 3 first column; OR = 1.8; 95% CI 1.1-3.1, for 30 minutes; OR = 2.1; 95% CI 1.2-3.7, for 1 to 2 hours; OR = 2.0; 95% CI 1.2-3.5, for 3 and more hours daily listening to music).

**Table 2:** Frequency (%) of electronic-media use, stratified for type of headache.

	No headache (n = 536)	Any headache (n = 489)	Pure migraine (n = 42)	Pure TTH (n = 212)	Migraine+TTH (n = 122)	MH (n = 113)
**Mobile phone**						
No mobile phone	12.3	9.6	7.1	8.5	10.7	11.5
<5 minutes	66.2	65.4	61.9	68.4	67.2	59.3
6 - 15 minutes	14.4	17.0	21.4	17.9	18.0	12.4
16 - 30 minutes	3.9	5.1	4.8	2.8	4.1	10.6
30 minutes or more	3.2	2.9	4.8	2.4	0.0	6.2
*p†*		*0.1847*	*0.1572*	*0.6685*	*0.6295*	***0.0171***

**Computer/internet**						
Never	14.6	11.7	14.3	9.9	8.2	17.7
30 minutes	34.0	33.7	26.2	35.4	38.5	28.3
1 - 2 hours	36.0	40.7	47.6	40.6	39.3	39.8
3 hours or more	15.5	13.9	11.9	14.2	13.9	14.2
*p*		*0.4306*	*0.7487*	*0.3721*	*0.4681*	*0.8360*

**Television/videos**						
Never	5.2	5.5	2.4	4.2	9.8	4.4
30 minutes	27.8	31.9	28.6	36.3	32.8	23.9
1 - 2 hours	52.4	50.3	50.0	50.5	43.4	57.5
3 hours or more	14.6	12.3	19.0	9.0	13.9	14.2
*p*		*0.1406*	*0.4388*	***0.0445***	*0.0573*	*0.5122*

**Game consoles**						
Never	71.6	74.0	71.4	75.9	77.0	68.1
30 minutes	16.0	16.8	19.0	17.9	15.6	15.0
1 - 2 hours	9.3	7.4	7.1	4.7	7.4	12.4
3 hours or more	3.0	1.8	2.4	1.4	0.0	4.4
*p*		*0.1540*	*0.7987*	***0.0458***	*0.0777*	*0.2540*

**Music**						
Never	11.0	5.1	2.4	6.1	3.3	6.2
30 minutes	30.8	27.8	31.0	25.0	28.7	31.0
1 - 2 hours	27.4	32.9	21.4	36.9	37.7	24.8
3 hours or more	30.8	34.2	45.2	32.1	30.3	38.1
*p*		***0.0026***	***0.0493***	***0.0341***	*0.0817*	*0.1064*

**Overall media index‡**						
Never	1.5	0.8	0.0	0.5	0.8	1.8
30 minutes	1.9	4.1	4.8	3.3	4.9	4.4
1 - 2 hours	27.4	29.2	26.2	28.3	36.1	24.8
3 - 4 hours	31.2	33.5	33.3	38.7	26.2	31.9
5 - 6 hours	25.6	22.1	19.0	20.8	23.8	23.9
7 hours or more	12.5	10.2	16.7	8.5	8.2	13.3
*p*		*0.0725*	*0.9217*	***0.1221***	***0.0369***	*0.7665*

† p values of Cochrane-Armitage tests for underlying trends of media use between no headache vs. different types of headache.

‡ The overall media index was estimated by adding up time spent with computer/internet, television/videos and game consoles.

**Table 3:** Adjusted odds ratios (95% confidence intervals) in comparison to no headache for occupation with electronic media.†

	Any headache (n = 489)	Pure migraine (n = 42)	Pure TTH (n = 212)	Migraine+TTH (n = 122)	MH (n = 113)
**Mobile phone**					
No mobile phone	1	1	1	1	1
<5 minutes	1.1 (0.7-1.7)	1.1 (0.3-4.0)	1.3 (0.8-2.4)	0.9 (0.4-1.7)	0.9 (0.5-1.7)
6 - 15 minutes	1.2 (0.7-2.0)	1.7 (0.4-7.0)	1.3 (0.6-2.6)	1.2 (0.5-2.7)	0.8 (0.3-1.8)
16 - 30 minutes	1.0 (0.4-2.2)	0.5 (0.1-4.8)	0.6 (0.2-2.1)	0.9 (0.2-3.6)	1.5 (0.5-4.4)
30 minutes or more	0.7 (0.3-1.7)	0.9 (0.1-7.9)	0.7 (0.2-2.4)	-	1.1 (0.3-3.8)

**Computer/internet**					
Never	1	1	1	1	1
30 minutes	1.2 (0.8-1.9)	0.8 (0.3-2.4)	1.3 (0.7-2.4)	**2.3 (1.1-5.1)**	0.8 (0.4-1.5)
1 - 2 hours	1.3 (0.9-2.0)	1.2 (0.4-3.4)	1.4 (0.8-2.6)	1.6 (0.7-3.5)	1.0 (0.5-1.8)
3 hours or more	1.2 (0.7-2.1)	0.6 (0.1-2.8)	1.5 (0.7-3.1)	1.9 (0.7-4.9)	1.0 (0.4-2.1)

**Television/videos**					
Never	1	1	1	1	1
30 minutes	1.1 (0.6-2.0)	1.7 (0.2-14.6)	1.7 (0.8-4.0)	0.7 (0.3-1.5)	1.0 (0.3-2.8)
1 - 2 hours	1.0 (0.5-1.7)	2.8 (0.2-22.9)	1.3 (0.6-3.0)	0.6 (0.3-1.2)	1.2 (0.4-3.2)
3 hours or more	0.9 (0.4-1.8)	3.3 (0.4-29.9)	0.8 (0.3-2.4)	0.7 (0.2-1.9)	1.0 (0.3-3.5)

**Game consoles**					
Never	1	1	1	1	1
30 minutes	1.4 (0.9-2.0)	2.2 (0.9-5.5)	1.4 (0.9-2.2)	1.3 (0.7-2.4)	1.3 (0.7-2.3)
1 - 2 hours	1.2 (0.8-2.0)	1.7 (0.4-6.7)	0.7 (0.3-1.4)	1.3 (0.6-3.0)	**2.4 (1.2-4.9)**
3 hours or more	1.0 (0.4-2.3)	1.2 (0.1-10.6)	0.7 (0.2-2.5)	-	2.7 (0.9-8.2)

**Music**					
Never	1	1	1	1	1
30 minutes	**1.8 (1.1-3.1)**	4.0 (0.5-31.8)	1.4 (0.7-2.7)	2.7 (0.9-8.0)	1.8 (0.7-4.3)
1 - 2 hours	**2.1 (1.2-3.7)**	1.9 (0.2-16.6)	**2.0 (1.0-4.0)**	**3.8 (1.3-11.1)**	1.4 (0.6-3.6)
3 hours or more	**2.0 (1.2-3.5)**	5.0 (0.6-39.6)	1.7 (0.8-3.4)	2.3 (0.7-7.1)	2.2 (0.9-5.4)

**Overall media index‡**					
Max. 30 minutes	1	1	1	1	1
1 - 2 hours	0.7 (0.4-1.3)	0.6 (0.1-3.4)	0.7 (0.3-1.9)	0.8 (0.3-2.0)	0.5 (0.2-1.4)
3 - 4 hours	0.8 (0.4-1.6)	0.6 (0.1-3.2)	1.1 (0.4-2.6)	0.6 (0.2-1.6)	0.6 (0.2-1.7)
5 - 6 hours	0.6 (0.3-1.3)	0.8 (0.1-4.4)	0.6 (0.3-1.7)	0.8 (0.3-2.2)	0.6 (0.2-1.7)
7 hours or more	0.7 (0.3-1.5)	0.6 (0.1-3.7)	0.7 (0.3-2.1)	0.5 (0.1-1.6)	0.8 (0.3-2.5)

† Logistic regression models were adjusted for age group, sex, family condition and socio-economic status.

‡ The overall media index was estimated by adding up time spent with computer/internet, television/videos and game consoles.

However, stratified for type of headache, statistically significant associations were rare and appeared to be rather unsystematic (Table 3): Listening to music for 1 to 2 hours was associated with higher risk for TTH (OR = 2.0; 95% CI 1.0-4.0) and migraine+TTH (OR = 3.8; 95% CI 1.3-11.1), usage of computer/internet for up to 30 minutes was associated with higher risk for migraine+TTH (OR = 2.3; 95% CI 1.1-5.1), and playing with game consoles for 1 to 2 hours was associated with higher risk for MH (OR = 2.4; 95% CI 1.2-4.9). No statistically significant effect with the overall media index was revealed.

Additional cross-adjustments for all types of eletronic media in one logistic regression model revealed no associations between any media and any type of headache (data not shown). These models suggest only significant associations with socio-demographic confounding variables, particularly with sex.

## Discussion

The aim of the present analysis was to investigate the association between use of electronic media and headache in a population-based sample of adolescents. While bivariate trend tests indicated that there might be differential associations between use of electronic media and prevalence of headache, most effects disappeared when the models were adjusted for socio-demographic variables. Only the associations between frequent listening to music and overall prevalent headache remained significant after adjustment, but this association was no longer seen if the influence of other electronic media was also considered. Thus, specific headache-related effects of frequency of calling with mobile phones, working with computers, watching television/videos or playing with game consoles were not found.

An adverse effect of high use of music on headache was also found by Zarowski et al., who reported that listening to music during nighttime may affect sleep habits and may, therefore, be associated with headache in children and adolescents [32]. However, sleeping habits have not been studied in the present investigation and we are, therefore, not able to test this more in detail. Also the effect of frequent listening to music was not significant after adjusting for the other variables. Furthermore, it can not be concluded whether the habit of "listening to music" is the consequence of frequent headaches, e.g. in the sense a self-therapy by relaxation, or the cause, e.g. in the sense of an additional stressor.

The majority of studies investigating associations between use of electronic media and headache focused on computer or mobile phone use. Most of them found adverse associations for frequent use of computers and both migraine and TTH [27-30]; only the study by Smith et al. did not find a statistically significant effect [31]. In a recent review on health complaints due to mobile phone use [15] results were controversial: While two studies found a statistically significant association between exposure to mobile phones and headache [[22,23]; see also [5,24]], two other studies did not [25,41]. In a double-blind provocation study no evidence was found that radio frequency fields from mobile phones cause headache [26]. With our present investigation, we also cannot confirm that usage of computer/internet or frequent calling with mobile phones could have an impact on headache.

Up to now we cannot exclude whether some of the isolated effects between specific electronic media and different types of headache (see Table 3) might result from multiple testing. This might especially be the case, as no stringent dose-response effects were observed for any electronic media. These inconsistent results can, therefore, not be regarded as 'true' associations. This has to be investigated in future studies.

### Strengths and limitations

The population-based data collection procedure and the application of multiple logistic regression models adjusting for relevant socio-demographic confounders are the major strengths of our investigation. Furthermore, we were able to involve the average duration of use of different types of electronic media within one study and could, therefore, create an overall media index and adjust for the various media types simultaneously.

One limitation of our study was the classification of headache, which was only based on self-reports. However, items of the used headache questionnaire in the present study were based on recently published ICHD-II criteria and were established and validated in comparable studies [40]. Furthermore, prevalences of migraine and TTH in the present study correspond quite well with estimates in other recent studies on headache in adolescents in Europe [18,19] and, especially, in Germany [20,21].

In addition, data on media use were based on self-reports, not on objective exposure measurements. Differential misclassification of the exposure (i.e. use of electronic media) or the outcome (i.e. headache) might have been an issue, as reported in studies on awareness bias [42]. However, no stringent statistically significant associations between media use and different types of headache were found. Thus, this type of potential information bias does not interfere with our conclusions.

The use of electronic media in the present study population was different in some aspects in comparison to the nationwide KiGGS study [3]: in the MobilEe study girls seem to use computer/internet more frequently, but music media less frequently than in the KiGGs study, and both girls and boys from the present study population seem to watch television/videos less frequently. These differences in the behavior of use of media reflect the finding that there are slightly more participants from families with higher levels of education [7,33]. To adjust for this variable, sex and SES were considered as confounders in the present study.

A power problem might have evolved when separate stratified analyses for different types of headache were done, simultaneously adjusting for socio-demographic confounding variables. Wide CIs, for example for the subgroup of participants with migraine, arose. However, also analyses comparing any vs. no headache, where no power problem could be observed, revealed no statistically significant effects of mobile phone calls, use of computer/internet, television/videos and game consoles.

With respect to the present objectives, a cross-sectional study might provide limited evidence. Corresponding to many other studies investigating associations between use of electronic media and headache, we conclude that the former might rather be treated as risk factors. However, we cannot exclude the possibility that subjects suffering from headache might have reduced their use of electronic media. Such behavior is not unlikely, since there is an ongoing public debate on adverse effects of use of media on health. To adjust for such effects which might have counterbalanced possible adverse effects of electronic media on headache, a longitudinal study which not only assesses the temporal sequence of electronic media use and onset of headache disorders, but also monitors age effects within groups with similar electronic-media use, might provide a better approach.

## Conclusions

In the present study, we observed only inconsistent associations between use of media and different types of headache. With respect to the current debate on adverse health effects of electronic-media use, we cannot point to systematic effects of single media types nor on specific types of headache which might predominantly be caused by the use of electronic media. To allow differentiation in the discussion of causes and effects and possible reverse causation, we suggest that these questions be investigated in a longitudinal study.

## Competing interests

This study is part of the project MobilEe: Mobilfunk - Exposition und Befinden (Mobile phone exposure and wellbeing in children and adolescents) which was funded by the German Mobile Telecommunication Research Programme. The authors declare that they have no competing interests.

## Authors' contributions

AMB was responsible for analysis and interpretation of data and the writing of the manuscript. RvK made contributions to the conception of the study and revised the manuscript for important intellectual content. ST was responsible for designing and conducting the study, was involved in analysis and interpretation of data and revised the manuscript for important intellectual content. SH was responsible for designing and conducting the study, was involved in analysis and interpretation of data and revised the manuscript for important intellectual content. AS revised the manuscript for important intellectual content. KR made contributions to conception and design and also to analysis and interpretation of data and revised the manuscript for important intellectual content. All authors read and approved the final manuscript.

## Pre-publication history

The pre-publication history for this paper can be accessed here:

http://www.biomedcentral.com/1471-2377/10/12/prepub

## References

[B1] Feierabend S Kutteroff A Medienumgang Jugendlicher in Deutschland [Media handling of adolescents in Germany] Media Perspektiven 2007 11 83 95

[B2] Feierabend S Kutteroff A Medien im Alltag Jugendlicher - multimedial und multifunktional [Daily media use among adolescents - multimedial and multifunctional] Media Perspektiven 2008 12 612 624

[B3] Lampert T Sygusch R Schlack R Nutzung elektronischer Medien im Jugendalter: Ergebnisse des Kinder- und Jugendgesundheitssurveys (KiGGS) [Use of electronic media in adolescence. Results of the German Health Interview and Examination Survey for Children and Adolescents (KiGGS)] Bundesgesundheitsbl - Gesundheitsforsch - Gesundheitsschutz 2007 50 643 652 10.1007/s00103-007-0225-7 17514448

[B4] Punamäki RL Wallenius M Nygard CH Saarni L Rimpelä A Use of information and communication technology (ICT) and perceived health in adolescence: the role of sleeping habits and waking-time tiredness J Adolesc 2007 30 569 585 10.1016/j.adolescence.2006.07.004 16979753

[B5] Söderqvist F Carlberg M Hardell L Use of wireless telephones and self-reported health symptoms: a population-based study among Swedish adolescents aged 15-19 years Environ Health 2008 7 18 10.1186/1476-069X-7-18 18495003 PMC2430957

[B6] Koivusilta LK Lintonen TP Rimpela AH Orientations in adolescent use of information and communication technology: a digital divide by sociodemographic background, educational career, and health Scand J Public Health 2007 35 95 103 10.1080/14034940600868721 17366093

[B7] Thomas S Heinrich S Kühnlein A Radon K The association between socioeconomic status and exposure to mobile telecommunication networks in children and adolescents Bioelectromagnetics 2009 31 20 27 10.1002/bem.20522 19598181

[B8] Robinson TN Reducing children's television viewing to prevent obesity: a randomized controlled trial JAMA 1999 282 1561 1567 10.1001/jama.282.16.1561 10546696

[B9] Hancox RJ Milne BJ Poulton R Association between child and adolescent television viewing and adult health: a longitudinal birth cohort study Lancet 2004 364 257 262 10.1016/S0140-6736(04)16675-0 15262103

[B10] Armstrong CA Sallis JF Alcaraz JE Kolody B McKenzie TL Hovell MF Children's television viewing, body fat, and physical fitness Am J Health Promot 1998 12 363 368 10182087 10.4278/0890-1171-12.6.363

[B11] Leena K Tomi L Arja RR Intensity of mobile phone use and health compromising behaviours--how is information and communication technology connected to health-related lifestyle in adolescence? J Adolesc 2005 28 35 47 10.1016/j.adolescence.2004.05.004 15683633

[B12] Vogelberg C Hirsch T Radon K Dressel H Windstetter D Weinmayr G Weiland SK von Mutius E Nowak D Leupold W Leisure time activity and new onset of wheezing during adolescence Eur Respir J 2007 30 672 676 10.1183/09031936.00152906 17596269

[B13] Russ SA Larson K Franke TM Halfon N Associations between media use and health in US children Acad Pediatr 2009 9 300 306 10.1016/j.acap.2009.04.006 19592321

[B14] Mathers M Canterford L Olds T Hesketh K Ridley K Wake M Electronic media use and adolescent health and well-being: cross-sectional community study Acad Pediatr 2009 9 307 314 10.1016/j.acap.2009.04.003 19592322

[B15] Seitz H Stinner D Eikmann T Herr C Röösli M Electromagnetic hypersensitivity (EHS) and subjective health complaints associated with electromagnetic fields of mobile phone communication--a literature review published between 2000 and 2004 Sci Total Environ 2005 349 45 55 10.1016/j.scitotenv.2005.05.009 15975631

[B16] Hutter HP Moshammer H Wallner P Kundi M Subjective symptoms, sleeping problems, and cognitive performance in subjects living near mobile phone base stations Occup Environ Med 2006 63 307 313 10.1136/oem.2005.020784 16621850 PMC2092490

[B17] Stovner LJ Hagen K Jensen R Katsarava Z Lipton R Scher A Steiner T Zwart JA The global burden of headache: a documentation of headache prevalence and disability worldwide Cephalalgia 2007 27 193 210 10.1111/j.1468-2982.2007.01288.x 17381554

[B18] Zwart JA Dyb G Holmen TL Stovner LJ Sand T The prevalence of migraine and tension-type headache among adolescents in Norway. The Nord-Trondelag Health Study (Head-HUNT-Youth), a large population-based epidemiological study Cephalalgia 2004 24 373 379 10.1111/j.1468-2982.2004.00680.x 15096226

[B19] Laurell K Larsson B Eeg-Olofsson O Prevalence of headache in Swedish schoolchildren, with a focus on tension-type headache Cephalalgia 2004 24 380 388 10.1111/j.1468-2982.2004.00681.x 15096227

[B20] Fendrich K Vennemann M Pfaffenrath V Evers S May A Berger K Hoffmann W Headache prevalence among adolescents--the German DMKG headache study Cephalalgia 2007 27 347 354 10.1111/j.1468-2982.2007.01289.x 17376112

[B21] Kröner-Herwig B Heinrich M Morris L Headache in German children and adolescents: a population-based epidemiological study Cephalalgia 2007 27 519 527 10.1111/j.1468-2982.2007.01319.x 17598791

[B22] Chia SE Chia HP Tan JS Prevalence of headache among handheld cellular telephone users in Singapore: a community study Environ Health Perspect 2000 108 1059 1062 10.2307/3434959 11102297 PMC1240163

[B23] Sandström M Wilen J Oftedal G Hansson Mild K Mobile phone use and subjective symptoms. Comparison of symptoms experienced by users of analogue and digital mobile phones Occup Med 2001 51 25 35 10.1093/occmed/51.1.25 11235824

[B24] Röösli M Moser M Baldini Y Meier M Braun-Fahrländer C Symptoms of ill health ascribed to electromagnetic field exposure--a questionnaire survey Int J Hyg Environ Health 2004 207 141 150 10.1078/1438-4639-00269 15031956

[B25] Koivisto M Haarala C Krause CM Revonsuo A Laine M Hämäläinen H GSM phone signal does not produce subjective symptoms Bioelectromagnetics 2001 22 212 215 10.1002/bem.41 11255218

[B26] Oftedal G Straume A Johnsson A Stovner LJ Mobile phone headache: a double blind, sham-controlled provocation study Cephalalgia 2007 27 447 455 10.1111/j.1468-2982.2007.01336.x 17359515

[B27] Bener A Uduman SA Qassimi EMA Khalaily G Sztriha L Kilpelainen H Obineche E Genetic and environmental factors associated with migraine in schoolchildren Headache 2000 40 152 157 10.1046/j.1526-4610.2000.00021.x 10759915

[B28] Rossi LN Cortinovis I Menegazzo L Brunelli G Bossi A Macchi M Classification criteria and distinction between migraine and tention-type headache in children Dev Med Child Neurol 2001 43 45 51 10.1017/S001216220100007X 11201423

[B29] Alexander LM Currie C Young people's computer use: Implications for health education Health Edu 2004 104 254 261 10.1108/09654280410546745

[B30] Oksanen A Metsähonkala L Anttila P Aromaa M Jäppilä E Viander S Salminen J Helenius H Sillanpää M Leisure activities in adolescents with headache Acta Paediatr 2005 94 609 615 10.1080/08035250410023331 16188751

[B31] Smith L Louw Q Crous L Grimmer-Somers K Prevalence of neck pain and headaches: impact of computer use and other associative factors Cephalalgia 2009 29 250 257 10.1111/j.1468-2982.2008.01714.x 19143770

[B32] Zarowski M Mlodzikowska-Albrecht J Steinborn B The sleep habits and sleep disorders in children with headache Adv Med Sci 2007 52 Suppl 1 194 196 18229663

[B33] Thomas S Kühnlein A Heinrich S Praml G von Kries R Radon K Exposure to mobile phone telecommunication networks assessed using personal dosimetry and well-being in children and adolescents: the German MobilEe-study Environ Health 2008 7 54 10.1186/1476-069X-7-54 18983641 PMC2614418

[B34] Kurth BM Kamtsiuris P Hölling H Schlaud M Dölle R Ellert U Kahl H Knopf H Lange M Mensink GB Neuhauser H Rosario AS Scheidt-Nave C Schenk L Schlack R Stolzenberg H Thamm M Thierfelder W Wolf U The challenge of comprehensive mapping children's health in a nation-wide health survey: design of the German KiGGS-Study BMC Public Health 2008 8 196 10.1186/1471-2458-8-196 18533019 PMC2442072

[B35] Ganzeboom HBG de Graaf PM Treiman DJ A Standard International Socio-Economic Index of Occupational Status Social Science Research 1992 21 1 56 10.1016/0049-089X(92)90017-B

[B36] Ganzeboom HBG de Graaf PM Treiman DJ Internationally comparable measures of occupational status for the 1988 International Standard Classification of Occupations Social Science Research 1996 25 201 239 10.1006/ssre.1996.0010

[B37] Milde-Busch A Heinrich S Thomas S Kühnlein A Radon K Straube A Bayer O von Kries R Quality of life in adolescents with headache--results from a population-based survey Cephalalgia in press 10.1177/0333102409354389 20511211

[B38] Milde-Busch A Boneberger A Heinrich S Thomas S Kühnlein A Radon K Straube A von Kries R Higher prevalence of psychopathological symptoms in adolescents with headache. A population-based cross-sectional survey Headache in press 20100300 10.1111/j.1526-4610.2009.01605.x

[B39] King A Wold B Tudor-Smith C Harel Y The health of youth. A cross-national survey WHO Reg Publ Eur Ser 1996 69 1 222 8756108

[B40] Headache Classification Subcommittee of the International Headache Society The International Classification of Headache Disorders Cephalalgia 2004 24 2 Suppl 1 9 160 14979299 10.1111/j.1468-2982.2003.00824.x

[B41] Santini R Seigne M Bonhomme-Faivre L Bouffet S Defrasne E Sage M Symptoms reported by mobile cellular telephone users Pathol Biol 2001 49 222 226 10.1016/S0369-8114(01)00132-8 11367556

[B42] Moffatt S Mulloli TP Bhopal R Foy C Phillimore P An exploration of awareness bias in two environmental epidemiology studies Epidemiology 2000 11 199 208 10.1097/00001648-200003000-00020 11021620

